# Perspectives of health care providers on the provision of intermittent preventive treatment in pregnancy in health facilities in Malawi

**DOI:** 10.1186/s12913-015-0986-x

**Published:** 2015-08-29

**Authors:** P. Stanley Yoder, Xavier Nsabagasani, Erin Eckert, Allisyn Moran, Yazoumé Yé

**Affiliations:** ICF International, Rockville, MD USA; Makerere University, Kampala, Uganda; Bureau for Global Health, USAID, Washington, DC USA

**Keywords:** Malawi, Malaria, IPTp, Antenatal care, Service providers

## Abstract

**Background:**

Nearly 20 years after the adoption by the government of Malawi of the provision of intermittent preventive treatment in pregnancy (IPTp) for malaria, only 55 % of pregnant women received at least two doses of sulfadoxine-pyrimethamine (SP) in 2010. Although several reasons for the low coverage have been suggested, few studies have examined the views of health care providers. This study examined the experiences of the nurses and midwives in providing antenatal care (ANC) services.

**Methods:**

This study was conducted in health facilities in Malawi that provide routine ANC services. Providers of ANC in Malawi were selected from in eight health care facilities of Malawi. Selected providers were interviewed using a semi-structured interview guide designed to address a series of themes related to their working conditions and their delivery of IPTp.

**Results:**

Nurses displayed detailed knowledge of ANC services and the rationale behind them. Nurses understood that they should provide two doses of IPTp during a pregnancy, but they did not agree on the timing of the doses. Nurses gave SP as directly observed therapy (DOT) at the clinic. Nurses did not give SP pills to women to take home with them because they did not trust that women would take the pills. Women who resisted taking SP explained they do not take drugs if they had not eaten, or they feared side effects, or they were not sick. Reasons for not giving the first or second dose of SP included a delay in the first ANC visit, testing positive for HIV, and presenting with malaria. None of the nurses were able to show any specific written guidelines on when to give SP. The challenges faced by the nurses include being overworked and persuading women to take SP under observation.

**Conclusion:**

The findings show that the nurses had gained the knowledge and technical skills to provide appropriate ANC services. With regard to IPTp, nurses need guidelines that would be available at the health facility about how and when to give SP. The adoption of the WHO guidelines and their diffusion to health care facilities could help increase the coverage of IPTp2 (at least two doses of sulfadoxine-pyrimethamine) in Malawi.

## Background

In countries of sub-Saharan Africa (SSA) where malaria is endemic, malaria during pregnancy has damaging effects on the health of mothers, the fetus, and neonates. The World Health Organization (WHO) has recommended a package of three interventions to address this danger: provide antimalarial drugs to prevent malaria during pregnancy, sleep under an insecticide-treated net (ITN), and provide prompt and effective treatment of malaria. Since the general recognition of the benefits of providing intermittent preventive treatment of malaria during pregnancy (IPTp) [1–3], many countries in SSA have sought to provide at least two doses of sulfadoxine-pyrimethamine (SP) as an integral part of antenatal care (ANC).

The WHO recommendations on IPTp have evolved over time as more research has shown the safety and efficacy of giving SP and its important contribution in the improvement of maternal and newborn health outcomes. The WHO recommendations of 2004 had stated that pregnant women should be given SP in the first two ANC visits after quickening [4]. In 2007 WHO recommended that pregnant women be given two or three doses of SP after the first trimester of gestation [5]. After further studies had demonstrated the safety of providing SP later in pregnancy, WHO revised its policy in 2012 to recommend that SP be given at every ANC visit after the first trimester up until the time of delivery with an interval of one month between doses, [6].

As countries in SSA adopt WHO guidelines and seek to implement them at national levels, they have set targets for coverage of at least two doses of IPTp and sought ways to assess their success. The indicator commonly used is the proportion of women who received at least two doses of SP after the first trimester during their last pregnancy. The target of African countries set in 2000 was to achieve 60 % coverage with IPTp2 by 2005, a target achieved by only The Gambia and Zambia [4]. The current target for IPTp2 coverage for all countries is 80 % by 2015. What challenges do countries face in reaching that target?

A meta-analysis of national survey data from 2009–2011 of IPTp2 coverage in 22 countries in SSA suggests that the low coverage of IPTp2 is not likely to be caused by low attendance of ANC clinics since in 20 of 22 countries, more than 60 % of pregnant women made at least two ANC visits [1]. An analysis of data on IPTp coverage from the Demographic and Health Surveys (DHS) in 16 countries in SSA concluded that the appropriate delivery of IPTp depends more on the dynamics of service delivery than on any differential access to ANC [7]. A recent review and meta-analysis of factors affecting the delivery, access, and use of interventions to prevent malaria in pregnancy in SSA found that barriers to the provision of IPTp were: 1) unclear policy and guidance on IPTp, 2) general health care system issues, (stock-outs and user fees), 3) poor quality of care, 4) misunderstanding by health care providers of protocols related to the timing of giving SP, and 5) low attendance at ANC clinics [8]. Health care provider confusion about the timing of the two doses of SP, and whether SP can be given on an empty stomach, featured prominently in many publications [2, 9–12].

A number of recent qualitative studies of health care providers’ experience in delivering IPTp to clients of ANC services have shown how working conditions and uncertainty about the timing of IPTp contribute to low coverage of IPTp [9, 13, 14]. Two recent studies of the experiences of health care providers in Nigeria found that the majority was confused about national IPTp policy on the timing of giving IPTp, and most of them did not give SP under directly observed therapy (DOT) [2, 10]. Interviews with health workers in the Ségou region of Mali found that the health care providers were confused about how and when to give SP [13].

### Antenatal care and IPTp in Malawi

The government of Malawi first adopted a policy of providing IPTp as part of comprehensive antenatal care in 1993. Ministry of Health guidelines of 1997 recommended that two doses of SP be given to pregnant women in ANC clinics: a first dose on the first ANC visit after the first trimester, and the second dose between 28 and 34 weeks (Ministry of Health and Population. National Malaria Policy Guidelines. 1997. Unpublished MOH policy document). The IPTp strategy was revised in 2002 and recommended that pregnant women be given two doses of SP after the first trimester at least one month apart under directly observed therapy (DOT) as part of ANC services.

The Ministry of Health guidelines for the provision of IPTp have changed over time in Malawi, but the various Ministry divisions did not always make the same recommendations. In 2006 the Ministry of Health adopted a policy of Focused Antenatal Care (FANC) as part of an essential health care package for maternal health (Ministry of Health. Focused Antenatal Care and Prevention of Malaria during Pregnancy. Training Manual for Healthcare Providers. 2006. Unpublished MOH policy document). The policy recommends that pregnant women make at least four ANC visits with the first visit in the first trimester of gestation. The policy guidelines for the provision of IPTp presented in the National Reproductive Health Strategy, 2006–2010 recommended that IPTp be given at 26 and 32 weeks of gestation (Ministry of Health. National Reproductive Health Service Delivery Guidelines. Reproductive Health Unit. 2007. Unpublished policy document). The National Malaria Treatment Guidelines, also published in 2007, recommended that at least three doses of SP be given at least four weeks apart after the first trimester (Ministry of Health. National Malaria Treatment Guidelines. Lilongwe: National Malaria Control Programme; 2007).

The US President’s Malaria Initiative (PMI) requested a review of Malaria in Pregnancy documents in late 2013 for 19 countries, including Malawi. This review examined government documents to assess the degree of coherence and consistency in the guidelines provided for giving SP. The review found that in Malawi, the guidelines were unclear and did not follow WHO recommendations regarding the timing of giving SP, in the designation of giving SP by specific weeks, and in prohibiting the provision of SP after 36 weeks of gestation [15]. The report also noted that the MOH of Malawi had formed a Technical Working Group to revise and standardize guidelines for Malaria in Pregnancy recommendations along the lines suggested by WHO.

In July of 2011 the Ministry began implementation of a new strategy to test and treat women who test positive for HIV in ANC clinics [16]. Known as Option B+, the strategy sought to provide testing, counseling, and anti-retroviral therapy (ART) for all HIV+ women within PMTCT no matter what their CD4 count. The strategy was generated and adopted in a workshop in April 2011 that involved government officials, private parties, and international donors [17]. All pregnant women attending ANC clinics are tested for HIV, and those who test positive for HIV are encouraged to enroll in ART services.

Data from the 2010 Malawi DHS showed that while 95 % of the women had made two or more ANC visits during the most recent pregnancy, the national IPTp2 coverage was 55 % [17]. Part of the reason that IPTp2 was not higher may be that most pregnant women in the sample who attended an ANC clinic at least once made their first visit after the first trimester: 48 % came first when they were 4–5 months pregnant, and 36 % at 6–7 months of pregnancy. The median number of months of gestation at the first visit was 5.6 months. Given the relatively high proportion of women who make at least two ANC visits during a pregnancy, low use of ANC services does not appear to be a factor in IPTp2 coverage in Malawi. Other possible explanations for relatively low coverage of IPTp2 are related to the process of providing SP within ANC clinics: the actions and understandings of nurses and midwives, or the knowledge and actions of women attending ANC services.

Ashwood-Smith and colleagues conducted a study of the provision of sulphadixine-pyrimethamine (SP) in pregnancy in Blantyre District of Malawi in 2001 [11]. The study found that the majority of the medical staff did not understand government guidelines clearly and were confused by the instructions to give the first dose during the second trimester and the second in the third trimester. Similarly, a study by Launiala and colleagues examined factors that affected compliance with IPTp guidelines by conducting focus groups and individual interviews among Yao women in Machinga district of southern Malawi. The study found that late enrolment in ANC clinics, unclear messages from nurses about taking SP, and the limited understanding of IPTp among women, all limited the uptake of SP in ANC clinics [12]. Our study covers topics similar to these studies, but places more emphasis on the context of service delivery in ANC clinics and on the perspectives of care providers on their own work.

## Methods

### Study design

This paper is derived from a slightly wider qualitative study of the experiences of health care providers who deliver antenatal services in health facilities in Malawi as described by the service providers themselves [18]. The paper focuses on the factors that affect the delivery of IPTp in the context of ANC services from the perspectives of the nurses and midwives who provide the services. The study examined the ways in which the training, the working conditions, the context of ANC delivery, and the knowledge of government guidelines for IPTp affected the delivery of SP to pregnant women in ANC clinics. The specific questions included:

- How do nurses and midwives describe the ANC services they routinely provide to pregnant women in ANC clinics?
- What do the nurses and midwives who provide antenatal services understand about the Ministry of Health policy on IPTp?
- What do they know about the purpose and importance of IPTp?
- What has been the experience of nurses and midwives in providing SP to pregnant women in the context of ANC services in general?

### Study context and setting

This study was designed to complement the data on ANC services collected by the 2013 Service Provision Assessment (SPA). The 2013-14 SPA in Malawi was developed as a census of all formal sector health facilities in the country, including hospitals, health centres, maternities, dispensaries, and health posts [19]. The survey included observation of the delivery of ANC services as well as exit interviews with ANC clients. MOH personnel who were managing the SPA supported this study by selecting the health care facilities to be visited, arranging for the actual visits, and introducing the person conducting the interviews to facility directors. Health care services in Malawi are provided by government health facilities and by facilities managed by the Christian Health Association of Malawi (CHAM). ANC is provided by nurses and midwives who work in specialized ANC clinics. Health care facilities provide PMTCT (prevention of mother-to-child transmission) services as part of standard ANC services.

### Ethical clearance

This study was an extension to the Service Provision Assessment (SPA) implemented in 2013 by the Ministry of Health (MOH) with technical assistance from ICF International. The SPA protocol was approved by the Institutional Review Board of ICF International. The SPA protocol was also submitted for ethical clearance by ICF to the Malawi National Health Sciences Research Committee. The committee determined that the Malawi SPA was a routine assessment, and therefore did not need any formal IRB clearance. At the level of data collection within health care facilities, after study objectives and methods were explained to potential respondents, informed content was obtained orally from each respondent before the interview began.

### Sample selection

The study assumed that ANC delivery might vary with the complexity of the medical services delivered, by whether the facility was administered by the government or by non-government entities, or by being located in urban or rural areas. The facilities for the study were therefore purposively chosen with those variables in mind. Eight health care facilities in the Central and Northern regions were visited. The facilities selected were two regional central hospitals, two district hospitals, and four rural hospitals. The non-government facilities visited were administered by CHAM.

In each facility, at least three health care providers who routinely provided ANC services were identified. The research specialist interviewed those three (or four) individuals with a semi-structured conversation guide. Table 1 shows the type of facility, the titles of those interviewed, and the specialized training each of the respondents reported [17]. Few respondents reported having taken any specialized training in IPTp service delivery.

**Table 1:** Type of health care facility and qualifications of respondents interviewed

Type of facility	Type of respondent	Qualifications
Regional Central Hospital Urban	Enrolled nurse and community nurse-midwife	ART, PMTCT, STI, IDSR, trauma for patients involved in car accidents
	Nurse and midwife technician	ART initiation, IPTp, Option B+
	Nurse and midwife	Care counseling, in-service on SP, infant feeding, nurses as managers, PMTCT
Government Hospital Rural	Clinical officer	IPTp
	Community nurse and midwife	ART, CBMAM, focused ANC, IMCI, infection prevention, malaria case management, PMTCT, pneumonia case management
	Nurse and midwife technician	ART
CHAM Hospital Rural	Registered nurse and midwife	ART
	Nurse and midwife	
	Community nurse and midwife	FANC, SP
Government Hospital Rural	Community health nurse	Family planning, infection prevention, supportive supervision
	Community health nurse	FANC, HIV/AIDS counseling and testing, PMCT
	Community health nurse	ART , HIV/AIDS counseling and testing
CHAM Hospital Rural	Community nurse technician	ARV, counseling, family planning, FANC, PMTCT, SP, tuberculosis management
	Senior community health nurse	
	Nurse midwife	ART
Regional Central Hospital Urban	Nurse and midwife technician	Family planning, FANC, infection prevention reproductive health, VIA
	Nurse and midwife technician	
	Nurse and midwife technician	
	Nurse and midwife technician	FANC, PMTCT, STI, VIA
Government Hospital Semi-urban	Enrolled community nurse and midwife	
	Community nurse and midwife	
	Community health nurse	ARI, ART, CMAM, community mobilization, ETAT, FANC, integrated community-based maternal and neonatal health, PMTCT
Government Hospital Rural	Enrolled midwife technician	
	Nurse and midwife technician	FANC, intensive training in HTC, PMTCT, safe motherhood
	Nurse and midwife technician	
	Nurse and midwife technician	IMCI, integrated ART, PMTCT, youth-friendly services

*ANC* antenatal care, *ARI* acute respiratory infection, *ART* antiretroviral therapy, *ARV* antiretroviral, *CBMAM* community based management of acute malnutrition, *CMAM* community management of acute malnutrition, *ETAT* emergency triage and assessment treatment, *FANC* focused antenatal care, *HTC* HIV testing and counseling, *IDSR* integrated disease surveillance and Response, *IMCI* integrated management of childhood illnesses, *IPTp* intermittent preventive treatment in pregnancy, *PMTCT* prevention of mother-to-child transmission, *SP* sulfadoxine-pyrimethamine, *STI* sexually transmitted infection

### Data collection and analysis

A total of 26 interviews were conducted in eight health facilities in July and August, 2013. In all cases, the three or four interviews were conducted during a one-day visit to a health care facility. All but one of the respondents held the title of nurse or midwife, or both. The exception was a clinical officer interviewed at a rural government facility. Since those with the titles of nurse, of community health nurse, or of midwife all had similar training as nurses, we will most often refer to them in this text simply as nurses.

The interviews were guided by a semi-structured conversation guide that focused on: 1) the professional training received; 2) working conditions and responsibilities within the facility; 3) their knowledge of the rationale for ANC services, 4) their knowledge of MOH policy on IPTp, and 5) their experience in providing IPTp as part of antenatal care. The conversation guide was pre-tested in a health facility in Lilongwe and then revised to eliminate repetitions and streamline the questioning process. Interviews were conducted in English and recorded with permission of the respondents (two declined), then transcribed and typed for analysis.

Transcripts were coded manually according to the themes cited above. The specific topics mentioned under each theme as well as the relative importance given to each topic were noted as part of content analysis. Summaries of the accounts of respondents related to each theme were written to facilitate direct comparison among the accounts. The amount of variation in the topics discussed by the respondents was examined to identify the extent of consistency among the accounts. Special attention was paid to descriptions of the challenges they faced in persuading women to take SP at the clinic through direct observed therapy (DOT).

## Results

### Professional training, knowledge and work responsibilities

Most of the respondents had been trained in comprehensive nursing and midwifery, and nearly half had undertaken training in community health. A few had been trained away from station for a few days in the prevention of mother to child transmission of HIV (PMTCT), integrated management of childhood illnesses (IMCI), or anti-retroviral therapy (ART). Only two of the respondents reported that they had received special training in the provision of IPTp.

The nurses, midwives, and clinical officers working in these health facilities had all been trained to work in any department of a hospital in addition to providing ANC services; they indicated that they routinely worked in various departments of the facility. The nurses in all the facilities visited expressed concern about staff shortages and heavy workloads that leaves them exhausted. They reported that such arrangements lead to staff shortages in ANC clinics. A nurse in a rural facility expressed frustration about her multiple responsibilities:

*Because I am a community health nurse, I am supposed to work in the community, but in my job description I am supposed to work anywhere. I can go to the male ward; I can go to the female ward. I can go to the labor ward to conduct deliveries.*

The consistency in the knowledge shown by each respondent suggests that they were well trained as nurses in the same curriculum overall. It should also be noted that they spoke of the work of service provider with enthusiasm and with a concern for the interests of their clients, especially those with high risk pregnancies. They stressed the importance of giving a thorough ‘head-to-toe examination’ of ANC clients to identify risky pregnancies and to discover any abnormality that could be addressed. And they provided details about their efforts to persuade women to accept ANC services, especially ART and IPTp. They spoke of the difficulties some women had to overcome to attend ANC services: traveling long distances, a disinterested husband; a young woman very shy about being examined. They seemed sincere about wanting to perform well despite often serving for long hours.

### Nature of antenatal care services

The health care facilities contacted for this study each offered ANC services five days a week in a unit separate from other services. The rural facilities had fewer staff per client, less space, and fewer supplies than did the urban facilities. ANC services in these hospitals are ostensibly free except in the two central regional hospitals where women pay a fee (about 2.50 US dollars) unless they had been referred to that facility by another health care entity. One CHAM facility was charging a fee to mothers who came from outside its catchment area.

The reports of nurses on the services they provide as part of ANC varied little in their content. The ANC services mentioned include a physical examination, counseling and screening, basic tests, preventive services, and medications. Blood pressure and height and weight are recorded, and blood and urine are taken for basic tests. Test results as well as services received are recorded in a booklet women have purchased or been given called a “health passport” that serves as a record of their contacts with health services. The preventive services include tetanus toxoid vaccines, a drug for intestinal parasites, screening for anemia and the delivery of iron supplements, counseling and testing for HIV, and IPTp. Women are counseled about the danger signs of complicated pregnancy, about protecting themselves against HIV transmission, and about the benefits of taking sulfadoxine-pyrimethamine (SP). Women considered especially vulnerable for high risk pregnancies receive special attention: women younger than 18, those who are very short, those who have had any sort of an operation, and those with a physical handicap.

The nurses explained that the two ANC services that demand the most time and attention are PMTCT and the “head-to-toe” physical examination. HIV testing requires test kits and other equipment as well as time for individual counseling on the prevention of HIV transmission and the revelation of test results to others. Women who test positive for HIV also need to be counseled to join the ART program offered by the facility. As for the physical exams, they simply take time to complete. One nurse reported that she could conduct a total of 10 such examinations in one day. While the head-to-toe exams take time, both nurses and pregnant women value them highly.

The physical examination called head-to-toe is usually done on the first ANC visit, and is used to identify women with high risk pregnancies as well as check for any signs of health problems or abnormalities. The reasons for conducting a head to toe examination were explained by a midwife in a rural facility:

***Respondent:****Because when you do a head to toe examination, it is like you have assessed the whole person, and it is where you find the problems and you address them. Like when you are doing a head to toe examination you can see if the woman is healthy; if the woman is anemic, then you address those issues. Or like how big is the fetus in relation to the weeks that the mother is saying.*

The nurses interviewed displayed extensive knowledge about the antenatal services they delivered and the rationale for each one. For example, they explained why they gave albendazole for intestinal parasites; they knew about the importance of screening for anemia and giving iron supplements; they spoke about tetanus toxoid vaccine and when it should be given; they identified the tests that would be conducted with the blood and urine samples collected as part of the clinical examination. They seemed totally familiar with the routine to follow in counseling and testing for HIV, and for persuading women to enroll in ART if found positive for HIV infection.

Nurses begin each ANC clinic with a health education session for the women and for any spouses who may have come with their wives. To encourage male involvement in ANC, women who come with their spouses are served first. The prevention of malaria and the benefits of SP may be discussed in the health talk. A nurse from a rural hospital described their health talks related to malaria prevention in these terms:

***Interviewer:****So who gives the general health talks?*

***Respondent:****Ourselves.*

***Interviewer:****How do you find the talks?*

***Respondent:****How do I find the talk? The talk begins like a malaria talk; you say, ‘we are going to talk about malaria.’ Then we start with malaria, general signs of malaria, what do you do. What are symptoms? What are you given when you come and found with malaria? Then they say if you come when you are not pregnant they give you quinine or AL Then after that how can we prevent malaria? So in that topic how can you prevent malaria is when we introduce that component of SP.*

***…***

***Interviewer:****What do you find interesting in this whole arrangement of teaching people about SP?*

***Respondent:****What I find interesting is that most of the people already know. They already have the information about malaria, and when you teach them you get more information from them. So it is easy, they understand.*

### Knowledge of the rationale for IPTp

Respondents considered IPTp as a very important service delivered within ANC. When asked to rank their services by importance, the nurses ranked PMTCT first and IPTp second even though they said that all services were equally important. The nurses all knew that SP is given to prevent malaria during pregnancy, and that malaria during pregnancy can cause health problems for mother and fetus or neonate. Some nurses reported that malaria can cause anemia or premature delivery. A nurse in one rural health facility located in an area of high malaria prevalence said that preventing malaria in pregnancy was critical. Speaking of SP, she stated:

*It helps because without SP usually here, especially in this malaria area, there are so many mosquitoes. So they catch up on malaria so easily when they are pregnant.…Malaria can cause premature delivery, and it can cause anemia.*

Several nurses explained why SP is given as a prophylaxis; most of them mentioned protecting the mother and child from malaria, which could lead to miscarriage or still births. A nurse in an urban facility commented on giving SP in this manner:

***Interviewer:****During your diploma training they told you about SP?*

***Respondent:****Yes*

***Interviewer:****Tell me exactly what.*

***Respondent:****Fansidar (SP) is an antimalarial drug given to the antenatal mothers. And Fansidar is given usually in second trimester and the mother should have a fetal movement. That is when we can give Fansidar. Starting from 16 weeks above and second dose is given 28 to 32 weeks.*

***Interviewer:****28 weeks. So why do they give Fansidar in particular?*

***Respondent:****Malaria is a major problem for these mothers. It may lead into abortion or critical conditions like becoming very sick, and which we do not want mothers to live in that state. As a result we are trying to prevent it. That is why we are giving this Fansidar.*

The interviews showed that nurses were well informed about what constitute a dose of SP, about the need for giving two doses of SP, and about the negative consequences of malaria in pregnancy. On the other hand, divergences in views were found in the timing of IPTp, in what guidelines they were to follow, and in the ways to insist on giving SP as DOT.

### Understanding of ministry of health guidelines

The respondents were asked about the guidelines they followed to provide ANC services and to know how and when SP should be delivered. A community health nurse in a rural hospital explained that they have general guidelines for reproductive health services and for PMTCT, but the guidelines were not available in the facility. Nurses in one hospital were certain that guidelines for malaria prevention existed, but they were not certain where they were located. No nurse was able to show guidelines for how IPTp should be delivered in their place of work. Some nurses reported that they had been told about IPTp by their colleagues or their supervisor.

Nurses reported that they were trying to follow MOH guidelines on services, but the guidelines available were not adequate. They said that PMTCT and IPTp were two domains of services that were relatively new to them, and that they lacked direction in how to proceed with them both. They also expressed concerns about their inability to follow changes in the policies of the MOH as they occur. Most of the nurses indicated that the posters on the walls of the health facility showed guidelines for the provision of ANC services, but they were not sufficiently detailed to provide direction. A few had typed out some of the instructions gleaned from the posters and hung those instructions on their wall. Several ANC units had a few points from the guidelines posted on the walls that had been typed out by nurses as reminders of how to proceed.

Most of the nurses providing IPTp services understood that SP was not to be given during the first trimester, vaguely defined, and not after 36 weeks of gestation. For most, the first dose was to be given at 16 weeks (or 18 or 20), or after “quickening,” when a woman becomes aware of fetal movements. The second dose would then be given after four weeks or more. A few thought SP could be given during the first trimester; two mentioned they give the first dose of SP at 20 weeks. In any case, they knew that government policy stipulates that they give SP under directly observed therapy (DOT).

According to accounts, some nurses rely on quickening more than on the number of weeks of gestation. A nurse in an urban hospital explained that after the head-to-toe examination,

***Respondent:****… If nothing is detected, then you declare that particular person to be normal. And then you say: Any fetal movement? If she says no, you do not give (SP) even if she is 5 months along. If she has noticed fetal movement, you do give. And the drugs will be given by another nurse outside where she will be explained and usually Fansidar is given as DOT right there.*

***Interviewer:****What do you mean a dot?*

***Respondent:****Directly observed (laughs)*

***Interviewer:****Directly observed, why?*

***Respondent:****Most mothers cannot really manage to take Fansidar at their homes so we want them to take it right there.*

***Interviewer:****So mothers cannot take the Fansidar at their homes? Why do you think?*

***Respondent:****Most of them do not like the drugs. They are bitter and they say, I do not feel comfortable what, what. But nowadays they are used to it when we explain to them.*

A nurse in a rural hospital explained her thoughts on the dosage of SP:

***Respondent:****We give SP to pregnant women not because they are suffering from malaria but as a preventive measure. So they get two doses of SP during pregnancy.*

***Interviewer:****Two doses means what?*

***Respondent:****Two doses: the first one at 18 weeks; we give three tablets, and then another one before the end of 36 weeks.*

Most nurses stated that they would not give SP after 36 weeks of gestation, though one nurse said the upper limit was 30 weeks. A nurse from a rural government hospital said that she expected they would soon be giving more than two doses, for she had heard that government policy had changed. They were waiting for official notification before changing their practice.

### Experiences in providing SP

Nurses all sought to persuade women to take the SP pills as DOT following MOH policy. They insisted that women take the pills while being observed, for if a dose of SP is given to women to take at home, the nurses do not expect that the pills will be taken. As a nurse in a rural hospital explained:

***Respondent:****Yes, we first explain. Because they will always ask, ‘why am I taking this?’ We first explain: ‘you are taking this because you are pregnant, you are prone to malaria. Malaria has a lot of complications to your pregnancy.’*

Nurses have learned from experience that some women resist taking SP in the clinic. Women have told nurses that the pills are hard to swallow because they are large and bitter tasting. Some women say that SP has side effects, making them dizzy or nauseous. Others refuse the pills because they say they have not eaten and do not take drugs on an empty stomach. Still others state that they need not take any medication since they are not sick. The explanation below on how nurses proceed to give SP comes from a rural health facility, and is similar to many other explanations offered:

***Interviewer:****Good. So what are some of the challenges of giving SP?*

***Respondent:****Maybe the women are not willing to take it. Most of them refuse because they say, ‘We have not eaten anything,’ or ‘we are not sick.’ So earlier we were giving them pills to take home, but now we do DOT: directly observed therapy. So with DOT we have fewer problems because we observe the women taking it.*

Most of the nurses indicated that they understood the reluctance of the women to take SP. Several said that they themselves had trouble swallowing the pills when they were pregnant because of the bitter taste or that it made them dizzy. However, they explained that they would take the time necessary to convince women to take SP while in the ANC clinic rather than take at home. Several nurses stated that they encourage the women to return home quickly after taking SP so they can eat and drink as soon as possible.

The experience of nurses in providing SP to pregnant women taught them about the reasons women offered for initially refusing to take SP, and ways they could persuade women to accept the three white pills despite their bitter taste. Nurses recognized that women were not accustomed to taking drugs without food, and that some were afraid of possible side effects, mainly dizziness and nausea. Several nurses confessed that they too had trouble swallowing the pills when they were pregnant. The way the nurses spoke about the responses of women when they were asked to take SP indicated that they understood possible objections, but they still needed to persuade women to accept SP.

The practice of giving SP as described by respondents indicated that most of them seek to give two doses of SP to pregnant women between 16 weeks and 36 weeks of gestation with at least four weeks between doses. For a few nurses, the timing of the first dose was a few weeks later than 16 weeks, and the cut off period was a few weeks earlier than 36 weeks. They stated that women who made their first ANC visit in their seventh month would likely receive only one dose of SP.

## Discussion

The findings of this study show that in the facilities visited, the nurses have been well-trained to provide ANC services. They are well informed about the ANC services they provide, and they understand the rationale for the services. The study also noted the statements of nurses about their own dedication to their work as nurses and midwives, and their desire to follow Ministry policy for the services they provide. All the nurses also spoke about the problems of understaffing; those working in rural facilities mentioned shortages of materials and equipment for the standard provision of ANC services.

Given this positive picture of performance, and the fact that more than 90 % of pregnant women in Malawi in recent years have made at least two visits to an ANC clinic (Ministry of Health. National Malaria Treatment Guidelines. Lilongwe: National Malaria Control Programme; 2007), how is it that a substantial proportion of pregnant women have not been receiving the second dose of IPTp in Malawi? One reason for failure to receive a second dose seems to be the timing of the first ANC visit. According to the Malawi Demographic and Health Survey of 2010 (MDHS), only 12 % of pregnant women made an ANC visit during their first three months of pregnancy, and 36 % made that first visit during their sixth or seventh month [17]. Women who begin ANC visits in the seventh month of gestation have a short window of opportunity to make a second visit before 36 weeks of gestation, the cut off period mentioned by most nurses. This explanation for low prevalence of IPTp has also been noted in other studies of the provision of IPTp in Malawi [11, 12].

Certain circumstances can also lead to failure to receive SP. According to the nurses, a woman who is diagnosed as HIV positive will not be given SP, but will be invited to enroll in the anti-retroviral program. The MDHS of 2010 showed that among women with a live birth in the past three years who had attended ANC, 10 % were HIV positive. Women who present with malaria will be referred for treatment and will not receive SP. A small proportion of women will refuse to take SP and will not be persuaded by the nurses. Finally, it is logical to assume that some nurses fail to give a second dose for unspecified reasons when a woman is eligible for SP. Some of the nurses pointed out that they were sometimes unable to perform certain tests or provide services because necessary supplies and/or equipment were unavailable in the health facility.

Another contributing factor to the level of coverage of IPTp2 is the uncertainty among nurses about MOH guidelines for providing SP in ANC clinics. The nurses all understood that it was MOH policy to provide two doses of SP to pregnant women, but none of the ANC clinics visited displayed specific guidelines for giving IPTp. The policy of the National Malaria Control Programme to give three doses after the first trimester that dates from 2007 had clearly not reached these respondents. Most of them understood MOH policy to mean that they should give the first dose of SP at 16-20 weeks, or after quickening, and the second dose before 36 weeks. A few said they gave the first dose a bit later than 16-20 weeks, and no second dose after 30 weeks, indicating some uncertainty about the timing of the doses of SP. The lack of guidelines displayed in ANC clinics and the uncertainty of nurses about the timing of SP doses must contribute to a lack of consistency in giving a second or third dose of SP.

Part of the explanation for the lack of guidelines may be that guidelines from various units of the MOH have not provided the same instructions in the past for the timing of SP doses. The National Reproductive Health Service Delivery Guidelines of 2007 recommended that IPTp be given at 26 and 32 weeks of gestation (Ministry of Health. National Reproductive Health Service Delivery Guidelines. Reproductive Health Unit. 2007. Unpublished policy document). The National Malaria Treatment Guidelines of 2007 recommended that three doses of SP be given four weeks apart after the first trimester (Ministry of Health. National Malaria Treatment Guidelines. Lilongwe: National Malaria Control Programme; 2007). The Guide for the Management of Malaria of 2007 recommended that two doses of SP be given (Ministry of Health. Guide for the management of malaria. Lilongwe: National Malaria Control Programme; 2007). New guidelines can resolve those ambiguities.

### Limitations

The insights drawn from the study findings would have added significance had we been able to visit more than eight facilities. It would also have been useful to systematically interview the head or manager of each health facility to discuss their knowledge of MOH policy regarding the provision of antenatal care and guidelines for IPTp. We were, however, reassured by the consistency in the reports of respondents about their own training, their roles and responsibilities, the provision of ANC services, and their understanding of the practice of providing IPTp as part of ANC services.

## Conclusion

The accounts of the nurses on their provision of IPTp services, along with their understanding of MOH policy on IPTp and their description of the ANC services they routinely deliver, identify specific challenges in working toward the improvement of IPTp2 coverage. First, we should expect that understaffing in some facilities complicates the delivery of appropriate services. Second, some health care facilities in rural areas do not always have access to all the drugs and materials and equipment required to carry out all the tests recommended. Staff shortages are usually more severe in rural facilities. Third, and above all, having clear guidelines available in health facilities on the timing and manner of giving IPTp would facilitate the delivery of SP in a more standard and consistent manner. The lingering ambiguity and uncertainties about the timing of IPTp leads to variations in how and when SP is delivered.

At the time of the study (2013), Malawi had not yet adopted the WHO guidelines for IPTp, but the Ministry of Health has since adopted the WHO guidelines (2014). Those current guidelines indicate that SP can be given safely up until delivery, which increases the likelihood of giving at least two doses of SP to those who initiate ANC visits late in pregnancy. Training on those guidelines began in 2014. The adoption of the WHO guidelines, and their diffusion and discussion in all health care facilities in the country, can be expected to increase the coverage of IPTp2 in Malawi.
